# Ontogeny and caudal autotomy fracture planes in a large scincid lizard, *Egernia kingii*

**DOI:** 10.1038/s41598-022-10962-x

**Published:** 2022-04-29

**Authors:** James I. Barr, Catherine A. Boisvert, Kate Trinajstic, Philip W. Bateman

**Affiliations:** 1 School of Molecular and Life Sciences, Curtin University, Kent Street, Bentley, WA 6102 Australia; 2 CSIRO Health and Biosecurity, 147 Underwood Avenue, Floreat, WA 6014 Australia; 3 Curtin Health Innovation Institute, Curtin University, Kent Street, Bentley, WA 6102 Australia

**Keywords:** Developmental biology, Ecology, Evolution, Zoology

## Abstract

Many lizard species use caudal autotomy, the ability to self-amputate a portion of the tail, as an effective but costly survival strategy. However, as a lizard grows, its increased size may reduce predation risk allowing for less costly strategies (e.g., biting and clawing) to be used as the primary defence. The King’s skink (*Egernia kingii*) is a large scincid up to approximately 244 mm snout to vent length (SVL) in size when adult. Adults rely less on caudal autotomy than do juveniles due to their size and strength increase during maturation. It has been hypothesised that lower behavioural reliance on autotomy in adults is reflected in loss or restriction of caudal vertebrae fracture planes through ossification as caudal intra-vertebral fracture planes in some species ossify during ontogenetic growth. To test this, we used micro-CT to image the tails of a growth series of seven individuals of *E. kingii*. We show that fracture planes are not lost or restricted ontogenetically within *E. kingii*, with adults retaining between 39–44 autotomisable vertebrae following 5–6 non-autotomisable vertebrae. Even though mature *E. kingii* rely less on caudal autotomy than do juveniles, this research shows that they retain the maximum ability to autotomise their tails, providing a last resort option to avoid threats. The potential costs associated with retaining caudal autotomy are most likely mitigated through neurological control of autotomy and *E. kingii*’s longevity.

## Introduction

Autotomy, the self-induced loss of a body part, usually to avoid predation, is found across multiple taxa from invertebrates to vertebrates^1,2^. In lizards, the ability to shed a portion of their tail, providing a distraction to the predator and an opportunity for the lizard to escape, is one of the most familiar examples of autotomy^3–5^. Once autotomised, the tail regenerates over time, with the original bony vertebrae replaced by a rigid cartilaginous rod that lacks autotomy planes^6,7^. Although caudal autotomy is a very effective anti-predation strategy, how much of the tail is autotomised and the time and energy invested into regenerating the tail, can incur short- and long-term costs associated with locomotion, growth, reproduction and subsequent survival^8–12^. The fitness costs however, both short- and long-term, can potentially be mitigated in certain species depending on specific life-history characteristics and behavioural traits^12^.

Lizard species that possess intra-vertebral autotomy have pre-formed fracture planes within a series of tail vertebrae– the post-pygal vertebrae^13,14^. Preceding the post-pygal vertebrae within the tail are a series of non-autotomising vertebrae – the pygal vertebrae – that are associated with the attachment of the *caudifemoralis longus* muscle (CFL), as well as the muscles of the intromittent organs of males^15–19^. When relying on caudal autotomy to survive a threat, a lizard needs to drop a sufficient amount of its tail to distract the threat, but not so much that it incurs unnecessary costs – a balance known as the economy of autotomy^4,20^. As such, the number and position of caudal vertebrae with fracture planes will physically influence the effectiveness of caudal autotomy as an anti-predation strategy, the time and energy investment required to regenerate the tail, and the number of consecutive times caudal autotomy events can occur^4,5^. Phylogenetically the osteological features that allow for caudal autotomy can be lost or restricted within whole families or in species within families^5,13^. Although there are species and clades of lizards that cannot autotomise due to having tails with a specialised function (e.g., prehensility), there does not seem to be a clear phylogenetic pattern across lizards as a whole^13,14,21^.

Selective forces associated with predation risk can also lead to fracture planes being lost in certain species ontogenetically as the individual grows^4,5^. The degree in which fracture planes are lost varies across species from partial to complete loss^4,13^, and is likely influenced by the importance of the tail to each species as an adult^5,21^. Loss of fracture planes occurs through a fusion of the fracture plane within the caudal vertebrae, commencing dorsally from the neural arch extending ventrally toward the centrum, and extending proximally from the most distal vertebrae in the tail^13^. Predation risks faced by juvenile lizards are generally higher than those faced by adults; therefore, reliance on caudal autotomy is more adaptive for juveniles. For example, juveniles of certain species have evolved bright or contrasting colour patterns and/or different behaviours to that of adults to deflect attacks toward the tail, increasing the adaptiveness of caudal autotomy^22–25^. Conversely, adults show a greater ability to defend themselves physically against certain predators (e.g., some species of snake) due to their larger size and alternative defensive tactics^14,25–27^, avoiding costs associated with caudal autotomy.

The King’s skink (*Egernia kingii)* is a large skink endemic to the South-West of Western Australia and adjacent islands^28,29^. During ontogenetic development, *E. kingii* matures from small, gracile juveniles (7 g, SVL 60–80 mm) into large, robust adults (between 220 and 360 g, up to 244 mm SVL)^28,30,31^. Juvenile *E. kingii* are known to rely more heavily on caudal autotomy for survival than do adults^24^, with adults physically able to defend themselves against certain predators^26,32^. *Egernia kingii* belong to the lygosomine ‘Egernia group’, a small but diverse grouping of ~ 50 skink species across seven genera that have either lost their autotomy planes evolutionarily (*E. depressa* and *E. stokesii*) or ontogenetically (*Corucia zebrata*, *Tiliqua rugosa* and *T. scincoides*), or have retained autotomy planes as adults (*E. cunninghami, Liopholis inornata*, *T. gigas* and *T. nigrolutea*)^13,14,16,17^. The ‘Egernia group’ include both gracile and largely robust species ranging between 70-270 mm SVL as adults and have multiple species with different tail specialisations (e.g., *C. zebrata’s* semi prehensility, and *T. rugosa’s* fat storage)^29,33^. However, unlike some of the ‘Egernia group’ species, *E. kingii* is not known to have additional caudal specialisations beyond autotomy, and, as such, ontogenetic changes of caudal autotomy planes can be approached purely from an anti-predation perspective. Additionally, as *E. kingii* are a slow-maturing (3–5 years), long-lived (suspected ~ 20 years) species^30,33^, potential ossification of fracture planes has a longer ontogenetic time frame to occur compared to that of a short-lived species.

We predict that fracture planes would be lost or restricted in *Egernia kingii* as they age and change from gracile, autotomy-reliant animals to robust animals that are able to rely on other physical defence tactics^14^. In this study, we use 1) micro-computer tomography to investigate the caudal morphology of *E. kingii*, 2) identify the pattern and degree of fracture planes present, and 3) assess if the fracture planes are lost or restricted ontogenetically to certain regions of the tail. The use of a growth series in this context instead of comparing a single juvenile and adult individual allows for a more in-depth investigation of how ossification occurs ontogenetically over the lifespan of *E. kingii.* We provide valuable insight into how the importance of this anti-predation strategy changes morphologically during ontogeny, and discuss the potential implications of the change both within *E. kingii* and in other lizard taxa with alternative methods for balancing costs of anti-predation strategies to maximise their effectiveness.

## Results

**Figure 1:**
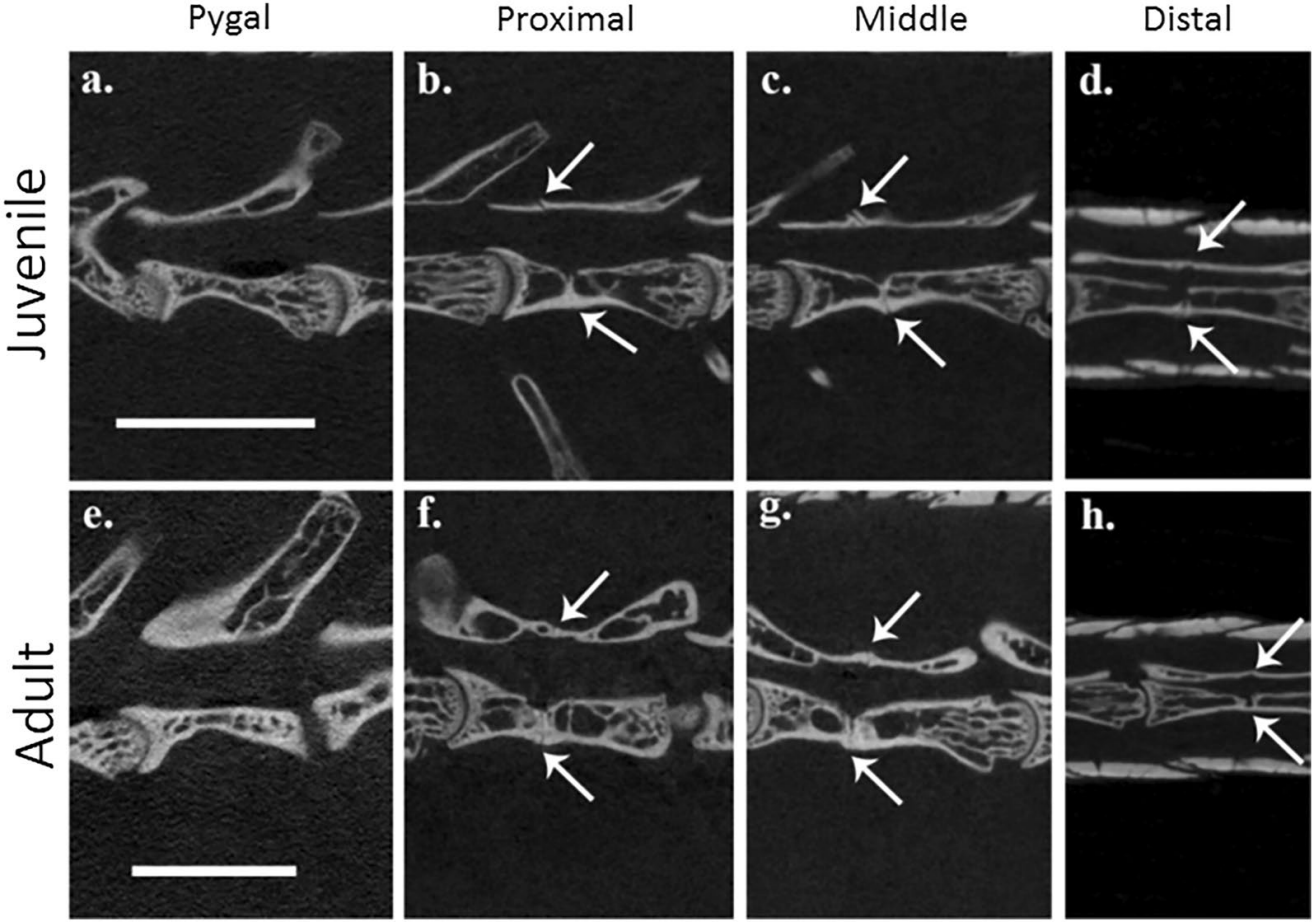
Sagittal longitudinal sections of *Egernia kingii* caudal vertebrae from reconstructed micro-CT images showing fracture planes (arrows) in the neural arch (top arrows) and centrum (bottom arrows). Images for a juvenile (top row) specimen (R62232) and an adult (bottom row) specimen (RI adult). Pygal vertebra without fracture planes and post-pygal vertebrae with fracture planes at proximal, middle, and distal positions of the tail (a., b., c., d. for the juvenile and e., f., g., h for the adult). White bar represents 5 mm for the individual specimens.

The micro-CT scans of the seven *E. kingii* specimens representing a growth series (SVL 132 mm–205 mm) reported in Table 1 show that intra-vertebral fracture planes were present in all specimens examined. Autotomy planes were not lost from, or restricted to, certain regions of the tail across the growth series, with fracture planes being evident in both the neural arch and centrum of the post-pygal vertebrae for all specimens examined (Fig. 1). The total number of post-pygal vertebrae ranged from 39—44 vertebrae, with an average (± SD) of 41 ± 2. The first 5–6 caudal vertebrae lacked fracture planes (pygal vertebrae), with the start of the post-pygal series of vertebrae (i.e., those with autotomy planes) at either the 6th or 7th caudal vertebra. The total number of caudal vertebrae in the *E. kingii* specimens ranged from 44 to 50, with an average (± SD) of 46 ± 2.

**Table 1:** Caudal vertebrae data for *Egernia kingii* specimens scanned by micro-CT.

Specimen #	Ontogenetic stage	SVL (mm)	TL (mm)	No. caudal vertebrae	No. pygal vertebrae	Caudal vertebrae # with first fracture plane	No. post-pygal vertebrae
R132059	Juvenile	132	172	47	6	7	41
R78064	Juvenile	145	195	47	6	7	41
R62232	Juvenile	150	185	44	5	6	39
R151388	Juvenile	165	229	50	6	7	44
R61436	Adult	185	226	46	5	6	41
R84662*	Adult	192	253	46	5	6	41
RI adult*	Adult	205	258	44	5	6	39

*R84662 tail was broken posthumously at caudal vertebrae vertebra #25, and RI adult tail was broken posthumously at caudal vertebra #9.

## Discussion

Ossification of fracture planes in the post-pygal vertebrae is the most common change associated with ontogenetic loss of autotomy in lizards^4,13,14^, and has been attributed to the greater relative importance of the tail after attaining a larger size, where tail loss would incur high costs to the individual^4,14^. Our results show that, for *E. kingii*, caudal autotomy fracture planes are not lost or restricted to a portion of the tail ontogenetically, with all specimens assessed from the ontogenetic growth series possessing intra-vertebral fracture planes throughout their post-pygal vertebrae. Although juvenile *E. kingii* rely heavily on caudal autotomy^24^, the complete retention of fracture planes and the ability to autotomise their tails as adults likely indicate that a net benefit from autotomy still applies to mature individuals^1,12,14,18^.

Caudal autotomy, although often thought of as primarily an anti-predation strategy, can be beneficial to survival for interactions with non-predatory threats and therefore be retained even in the absence of predators^34^. Both intra- and inter-specific aggressive interactions, often exacerbated in denser populations such as those occurring on islands, can result in tail loss in lizards for adults and juveniles^35–37^. For example, increased tail break frequencies in adult *Mediodactylus kotschyi* (Gekkonidae) was correlated with higher gecko abundance but not predator richness across island populations^34^. In *Anolis nebulosus* (Dactyloidae), increased frequencies of caudal autotomy were correlated with inter-and intra- species aggression for both adults and juveniles across mainland and island populations^35^. The distribution of *E. kingii* occurs throughout the South-West of Western Australia, including multiple offshore islands^28,29^. Some offshore islands, like Penguin Island, are free of terrestrial predators and *E. kingii* occur in high densities – up to 667–950 individuals/ha^38–40^. Non-predatory aggressive interactions are evident between adult lizards, especially during the mating period, and from nesting shorebirds (J. Barr, pers obs). Therefore, the retention of caudal autotomy in adults could be of potential benefit to individuals exposed to high levels of intra- or inter-specific aggression^5,34,41^.

For adult lizards, tail loss can have significant negative effects on the reproductive output of an individual. Tail loss can result in reduced mate attraction or mating success^42,43^, physical loss of caudal fat reserves vital in vitellogenesis and young development^9,44,45^, and decreased likelihood of reaching future reproductive seasons due to increased predation risk^46–48^. Tail loss during the reproductive season can reduce litter size, the number of young produced, and even skipping reproduction, especially in species that use their tails for fat storage^9,49^. Short-lived species have fewer reproductive seasons over their lifespan than long-lived species, particularly if they are missing their tail, and therefore rendered more vulnerable to predation. If autotomy occurs during the reproductive season, the reproductive output should be prioritised in short-lived species through energy investment into reproduction over regeneration, with long-lived species prioritising regeneration and survival for future reproductive seasons^8^. *Egernia kingii* is a slow-maturing (3–5 years), long-lived species, with a breeding season restricted to approximately two months in the Austral spring and summer^30,33^. Adults are likely to live upwards of 15–20 years, similar to the related *E. cunninghami*^33^, giving them a longer reproductive span compared to short-lived, fast-maturing species. Ontogenetic retention of caudal autotomy planes may allow *E. kingii* to balance the reproductive costs of caudal autotomy as adults through their longevity and future reproductive seasons, with retention of this nascent strategy serving as a last resort scenario and conveying an overall fitness benefit to the species^12^.

Behavioural decisions, including anti-predation strategies, are largely influenced by the perception or recognition of a threat^50–54^. Behavioural responses to threat perception and associated anti-predation behaviour can be learned or lost over time with exposure to or isolation from threats^54–58^. Both frequency and ease of caudal autotomy for lizards often increase in high threat environments^41,59^. Intra-vertebral caudal autotomy is under the conscious control of the individual, and active muscular contractions of the tail assist in breakage at the fracture plane, hence physical stimulus from a potential predator is not necessarily required^18,60,61^. The ability of the individual to regulate when and how much of its tail is autotomised may, especially within changing environments, confer a fitness benefit while minimising associated costs (e.g., dropping and regenerating a part of, as opposed to the entire tail) and hence the retention of caudal autotomy into adulthood.

Balancing the fitness costs and benefits of caudal autotomy in lizards has seen loss or restriction of fracture planes between clades and throughout ontogeny, with no clear phylogenetic pattern evident. Ontogenetic fitness costs, at least in intra-vertebral autotomising lizard species, may be mitigated through behavioural regulation and the species’ longevity. This would allow for the retention of caudal autotomy and its benefits as adults (e.g., to escape from potential threats if required) and reduce the potential long-term negative fitness effects, particularly those associated with reduced reproductive output. Here we show that caudal fracture planes are not lost or restricted during ontogenetic development of the large, long-lived skink *Egernia kingii* and suggest how fitness costs are likely to be balanced to allow for their retention. Additionally, the CT scanning analysis presented in this paper provides a non-destructive, powerful approach to examine in detail the fracture plane in a variety of preserved specimens. This approach will be very useful in examining the interaction between fracture plane loss and balancing the metabolic costs of regeneration.

## Methods

### Specimens

Seven preserved *E. kingii* specimens representing a juvenile to adult growth series (SVL 132 mm–205 mm) were chosen to investigate the ontogenetic change in caudal osteology and fracture planes. Six preserved *E. kingii* specimens came from the Western Australian Museum (WAM) (R61436, R62232, R78064, R84662, R132059 & R151388) and one recently deceased specimen from Rottnest Island (RI), donated by the Rottnest Island Authority (RIA). The specimen obtained from RIA was frozen at − 20 °C and preserved in 100% ethanol. Western Australian Museum specimens were formalin-fixed and stored in 70% ethanol. Specimens were selected based on the criteria of (1) being in good condition, (2) having their original tail intact (no regeneration) as determined from visual inspection, and (3) that preservation had not made specimen rigid, allowing easy manipulation during micro-CT scanning. Snout to vent length (SVL) and tail length (TL) of specimens was measured to the nearest mm using a flexible fabric measuring tape. The number of autotomisable vertebrae was obtained by visual confirmation of fracture plane presence from micro-CT images in the neural arch and centrum.

### CT scanning and analysis

Sample specimens were scanned individually using a SkyScan 1176 scanner (Bruker micro-CT, Kontich, Belgium) at the Centre for Microscopy, Characterisation and Analysis (CMCA), University of Western Australia. The CT scans were performed at 18 μm resolution (65 kV, 385µA, 300 ms, 1 mm Al filter, 0.5° rotation step, no frame averaging, 360° scan) producing 2000 * 1336-pixel images. Scanning images were reconstructed in NRecon v1.7.1.0 (Bruker micro-CT) using the modified Feldkamp cone-beam algorithm (Gaussian smoothing kernel (2), ring artefact correction (20), beam hardening correction (30%) and threshold for defect pixel masking (0–5%)). Three-dimensional models were constructed and manipulated in Avizo 2019.4 (Thermo Fisher Scientific). The caudal vertebral column of all specimens was examined using coronal and sagittal longitudinal sections from the Ortho Slice function, with the presence or absence of an autotomy plane in the centrum and neural arch marked as being present or absent (see Fig. 1).

## Data Availability

The datasets generated during and/or after the current study are available from the corresponding author on reasonable request.
